# Service evaluation for services for younger people with dementia in east locality of north Wales

**DOI:** 10.1192/bjo.2021.108

**Published:** 2021-06-18

**Authors:** Asha Dhandapani, Sathyan Soundararajan, Sharmi Bhattacharyya

**Affiliations:** BCUHB

## Abstract

**Aims:**

To evaluate Young-onset dementia (YOD) services in terms of referral, its appropriateness, time to diagnosis and other criteria as per protocol that we have adapted.

**Method:**

Case notes of those under 65 referred to Memory service for cognitive assessment between July 2017 and June 2018 were retrospectively reviewed to look at the time to diagnosis, appropriate referrals, post-diagnostic support, etc.

**Result:**

Compared to the previous evaluation, the number of patients referred to had increased from 47–48/ year earlier to 63/year. Only 1/3 were appropriate referral over the 10-year period whereas between 2017 and 2018 more than half were appropriate referrals. More than half of them were seen within 12 weeks of referral (35/63 available). Only 132/252 were diagnosed as having some form of dementia in the previous evaluation which was about 13 cases of YOD a year. In contrast, in our new evaluation 19 patients were diagnosed with some form of dementia. Inappropriate referrals had reduced by more than 50%. Appropriateness and timely referral had improved in this time frame.

**Conclusion:**

Dementia is considered ‘young onset’ when it affects people under 65 years of age. It is also referred to as ‘early onset’ or ‘working age’ dementia. However, this is an arbitrary age distinction that is becoming less relevant as increasingly services are realigned to focus on the person and the impact of the condition, not the age. Teaching sessions to educate primary & secondary care clinicians on appropriateness and timely referrals have helped in improving the care for patients with YOD. Services need to be developed further to be able to diagnose & support those with YOD. Repeat evaluations every year would help to inform improvement in quality & appropriateness of referrals.

